# Quantitative changes in gene transcription during induction of differentiation in porcine neural progenitor cells

**Published:** 2012-06-06

**Authors:** Jing Yang, Ping Gu, Steven Menges, Henry Klassen

**Affiliations:** 1 Gavin Herbert Eye Institute, University of California, Irvine, CA; 2 Department of Ophthalmology, Shanghai Ninth People's Hospital, School of Medicine, Shanghai Jiaotong University, Shanghai, China

## Abstract

**Purpose:**

Differentiation of neural stem/progenitor cells involves changes in the gene expression of these cells. Less clear is the extent to which incremental changes occur and the time course of such changes, particularly in non-rodents.

**Methods:**

Using porcine genome microarrays, we analyzed changes in the expression of 23,256 genes in porcine neural progenitor cells (pNPCs) subject to two established differentiation protocols. In addition, we performed sequential quantitative assessment of a defined transcription profile consisting of 15 progenitor- and lineage-associated genes following exposure to the same treatment protocols, to examine the temporal dynamics of phenotypic changes following induction of differentiation. Immunocytochemistry was also used to examine the expression of seven of these phenotypically important genes at the protein level. Initial primary isolates were passaged four times in proliferation medium containing 20 ng/ml epidermal growth factor (EGF) and 20 ng/ml basic fibroblast growth factor (bFGF) before differentiation was induced. Differentiation was induced by medium without EGF or bFGF and containing either 10 ng/ml ciliary neurotrophic factor or 10% fetal bovine serum (FBS). Cultures were fed every two days and harvested on days 0, 1, 3, and 5 for quantitative real-time PCR.

**Results:**

The microarray results illustrated and contrasted the global shifts in the porcine transcriptome associated with both treatment conditions. PCR confirmed dramatic upregulation of transcripts for myelin basic protein (up to 88 fold), claudin 11 (up to 32 fold), glial fibrillary acidic protein (GFAP; up to 26 fold), together with notable (>twofold) increases in message for microtubule associated protein 2 (*MAP2*) and C-X-C chemokine receptor type 4 (*CXCR4*), Janus kinase 1 (*Jak1*), signal transducer and activator of transcription 1 (*STAT1*), and signal transducer and activator of transcription 3 (*STAT3*). Transcripts for nestin and Krüppel-like factor 4 (*KLF4*) decreased sharply (>twofold). The specific dynamics of expression changes varied according to the transcript and treatment condition over the five days examined following induction. The magnitude of neuronal marker induction was greater for the ciliary neurotrophic factor condition while glial fibrillary acidic protein induction was greater for the FBS condition.

**Conclusions:**

The transient dynamic of *CXCR4* expression during induction of differentiation, as well as the upregulation of several major histocompatibility complex (MHC) transcripts, has implications in terms of graft integration and tolerance, respectively. These data confirm and extend in the pig the findings previously reported with murine retinal progenitors and support the use of this large animal model for translational development of regenerative approaches to neurologic diseases.

## Introduction

Neural progenitor cells (NPCs) are multipotent, tissue-specific cells that generate neuronal and glial cell types during development of the central nervous system [1,2]. As a consequence of the pivotal role of NPCs in development, the regulation of NPC behavior is as elaborate as it is important. Multiple molecular pathways have been implicated in the proliferation and differentiation of these cells, and at present, the topic remains incompletely understood. Considerable effort has been directed toward elucidating NPC differentiation in vivo using molecular genetic approaches in the mouse. The insights derived from those studies have been quite helpful to workers striving to direct the differentiation of pluripotent stem cells into tissue-specific lineages; however, results for cultured multipotent NPCs have been more limited. Nevertheless, the in vitro differentiation of cultured NPCs into neurons and glia remains an important assay for confirming multipotency and thus the identity of these cells.

Several methods for achieving NPC differentiation are commonly used; however, the methods themselves are rarely compared. For example, serum and mitogen withdrawal are recognized methods of inducing differentiation of NPCs, but suffer from a lack of specificity and poor viability, respectively. Examples of specific molecules known to influence NPC activity include fibroblast and epidermal growth factors, thyroid hormone [3], cortisol [4], retinoic acid [5], opioids [6], glutamate [7], as well as a wide range of neurotrophic factors [8], of which ciliary neurotrophic factor (CNTF) has attracted repeated attention as a putative differentiating agent [9,10].

CNTF is a 22-kDa protein that bears a familial relationship with leukemia inhibitory factor (LIF) and interleukin-6 (IL-6), all of which share a common amphipathic helical domain. In the central nervous system (CNS), CNTF is specifically expressed by astrocytes and has been shown to support the survival of all classes of peripheral nervous system neurons and many neurons from the CNS as well. In vitro, this neurotrophic factor has been associated with the induction of neurite outgrowth, promotion of the cholinergic phenotype in sympathetic neurons, and arrest of cell division in neuronal precursor cells [11,12] with various influences on the survival and differentiation of embryonic cells of neuronal and glial lineages [11,12]. In vivo, CNTF administration has been specifically associated with rescue of central neurons from axotomy-induced cell death and, under normal conditions, appears to play a general role in developing and maintaining the nervous system [13–15]. CNTF acts on glial progenitor cells from the neonatal rat optic nerve, which develop into either oligodendrocytes or type-2 astrocytes following exposure to this molecule [16]. A second role of CNTF regarding CNS glial cells is mitigating oligodendrocyte death in serum-free medium or upon exposure to tumor necrosis factor (TNF) [17].

Several major signal transduction pathways have been linked to neural development, one being activation of the Janus tyrosine kinase/signal transducer and activator of transcription (JAK/STAT) pathway by binding to glycoprotein 130 (gp130) [10,18] and others including the mitogen-activated protein kinase (MAPK) [14,19] and Notch [20] pathways. The CNTF receptor complex is known to include gp130 as a component and CNTF does signal through the JAK/STAT pathway [21], although signaling through additional pathways has also been reported, including the extracellular signal-related kinase (ERK) [22], phosphatidylinositol-3-kinase/AKT [23], MAPK, and Notch [24] pathways.

A variety of animal models have been used to investigate the effect of CNTF on central nervous system (CNS) progenitors, including mouse [19,25,26], rat [15,27,28], and chicken [29]. Rodent species and chicken embryos are generally preferred in the laboratory for several reasons, including availability of species-specific molecular reagents and relative ease of care and low cost of maintenance. More recently, however, the pig has assumed an increasingly prominent role as a large animal model for treating human disease [30], and this work has included studies of neural progenitor cell isolation [31] and transplantation to the retina [32,33]. The availability of porcine neural progenitor cells (pNPCs) now allows exploration of cultured NPC behavior in a large animal model and assessment of the influence of CNTF on cells of this species.

Genomic profiling has been used to characterize transcriptional patterns in NPCs in various research models [32,34,35]. In prior work, we employed conventional PCR, immunocytochemistry, flow cytometry, and microarrays using human-specific or cross-mammalian reagents and probes to examine the gene expression of pNPCs [31]. Subsequent work by another group used proteomics to examine the differentiation of cells of this type [36]. Recent progress in sequencing and annotating the porcine genome now permits the design of improved probes for use in this species. Here we use porcine DNA microarrays combined with quantitative real-time PCR and pig-specific primers to confirm and broaden considerably our initial findings for pNPCs in the context of standard proliferation conditions as well as fetal bovine serum (FBS) and CNTF-based differentiation conditions. Using these methods, we demonstrate global changes in transcription as well as dynamic alterations in the expression patterns of relevant phenotypic markers by cultured porcine NPCs as these cells transition from proliferative to more differentiated cell types.

## Methods

### Donor animals

Fetal pigs were harvested at 45 days gestational age from a crossbred adult sow under general anesthesia and the donor terminated without waking. The process was performed in accordance with the guidelines of the National Institutes of Health Guide for the care and use of laboratory animals (NIH Publications No. 80–23, revised 1978).

### Cell isolation and culture

Fetal pigs were transported to the laboratory on ice and dissected in a laminar flow hood approximately 8 h after removal from the maternal donor. The cranium was opened and the brain removed. Forebrains were minced mechanically, followed by digestion in 0.05% Trypsin Express (Invitrogen, Carlsbad, CA) for 5 min at 37 °C. Remaining tissue fragments were gently triturated using a 1 ml fire-polished glass Pasteur pipette to release single cells, and this process was repeated for two cycles. The resulting cell suspension was centrifuged at 1000 rpm for 5 min and resuspended in fresh culture medium comprised of Advanced Dulbecco's Modified Eagle Medium: Nutrient Mixture F-12 (D-MEM/F12; Invitrogen) with 2 mM GlutaMAX (Invitrogen,), N2 supplement (1%, Invitrogen), 20 ng/ml epidermal growth factor (EGF; Invitrogen), 20 ng/ml basic fibroblast growth factor (bFGF; Invitrogen), and 50 U/ml penicillin-streptomycin (Invitrogen). Cell viability was assessed with trypan blue (Sigma-Aldrich, St. Louis, MO), and cells were plated in uncoated 75 cm^2^ flasks at a cell density of 6.7×10^4^/cm^2^, followed by incubation at 37 °C under 5% CO_2_. Then 5% fetal bovine serum was included in the medium overnight to promote cellular viability and adherence, followed by a complete change to serum-free medium the next day. From that point onward, serum-free medium was used for culturing NPCs under proliferation conditions. Cells were fed by exchanging 90% of the medium for fresh medium every two days and passaged at 80% confluence, every four to five days, using 0.05% Trypsin Express.

### Differentiation conditions for neural progenitor cells

Early stage, passage-4 pNPCs were used as the starting point for the differentiation experiments. Cells in confluent T75 flasks were trypsinized and resuspended as a single-cell suspension (0.5×10^6^/ml), and then seeded into uncoated T75 flasks and allowed to grow for 48 h at 37 °C in serum-free standard medium (SM). The standard medium containing EGF and bFGF was then removed, and the cells were washed with basic DMEM/F12 medium without added growth factors. Cells were then changed to one of two different differentiation conditions, neither of which contained EGF or bFGF. These consisted of medium containing either CNTF (10 ng/ml, Chemicon, Temecula, CA) [11,12] or 10% FBS (Sigma-Aldrich). Controls were again maintained in standard proliferation medium containing EGF and bFGF. For all conditions, the medium was replaced twice during the five continuous days of the experiment. Images of the cultured cells were recorded using a Nikon inverted microscope, ECLIPSE TS100, with a Nikon DXM1200C camera (Nikon Instruments, Inc., Melville, NY).

### RNA extraction

Total RNA was extracted from three different groups of cells, the control group (grown in standard medium containing EGF and bFGF), the CNTF group (not containing EGF and bFGF), and the FBS group (not containing EGF and bFGF), and the samples were processed using an RNeasy Mini Kit (Qiagen, Valencia, CA) following the manufacturer’s instructions for samples obtained at experimental day 0, day 1, day 3, and day 5. RNA was quantified with spectrophotometer (ND-1000; NanoDrop Technologies Inc., Wilmington, DE) optical density (OD) absorption ratio OD260 nm/OD280 nm 2.00–2.10, OD260 nm/OD230 nm 2.00–2.20.

### Microarray analysis

All samples of total RNA from treatment day 5 were assessed for quality before processing by transferring a small amount of each sample (100 ng/well) onto an RNA Lab-on-a-Chip (Caliper Technologies Corp., Mountain View, CA) for evaluation via an Agilent Bioanalyzer 2100 (Agilent Technologies, Palo Alto, CA). Single-stranded, then double-stranded (ds), cDNA was synthesized from the poly(A)^+^ mRNA present in the isolated total RNA (5.0 μg total RNA starting material each sample reaction) using the SuperScript Double-Stranded cDNA Synthesis Kit (Invitrogen) and poly (T)-nucleotide primers that contained a sequence recognized by T7 RNA polymerase. A portion of the resulting ds cDNA was used as a template to generate biotin-tagged cRNA from an in vitro transcription reaction (IVT), using the BioArray HighYield RNA Transcript Labeling Kit (T7; Enzo Diagnostics, Inc., Farmingdale, NY). Approximately 15 μg of the resulting biotin-tagged cRNA was fragmented to strands of 35–200 bases long following prescribed protocols (Affymetrix GeneChip Expression Analysis Technical Manual, Santa Clara, CA). Subsequently, 10 μg of this fragmented target cRNA was hybridized at 45 °C with rotation for 16 h (Affymetrix GeneChip Hybridization Oven 640) to probe sets present on an Affymetrix GeneChip Porcine Genome Array. The GeneChip arrays were washed and then stained (SAPE, streptavidin-phycoerythrin) on an Affymetrix Fluidics Station 450, followed by scanning on an Affymetrix GeneChip Scanner 3000 7G. The results were normalized with the sketch-quantile method (Expression Console ver.1.1 software, Affymetrix, Inc.). Microarray data were then evaluated using JMP Genomics 4.1 (SAS Americas, Cary, NC). The data were analyzed with one-way ANOVA with a post hoc Student *t* test and the resulting p-values corrected using an false discovery rate (FDR) α<0.05 [37]. The resulting data table was annotated with reference to Tsai et al. [38]. JMP Genomics was also used to generate a principal component analysis, a Venn diagram, as well as a hierarchical cluster and heat map, using the default fast Ward’s method, in addition to volcano plots from the ANOVA results. Hierarchical clusters were analyzed using Database for Annotation, Visualization and Integrated Discovery (DAVID) Bioinformatics (Laboratory of Immunopathogenesis and Bioinformatics) to assess the types of genes present within each cluster [39,40]. Only clusters with an enrichment of p<0.05 were analyzed further.

### Real-time polymerase chain reaction assay

Selection of candidate markers was based on the results of our previous work with cells of this type, together with potential relevance to the current study. Particular emphasis was placed on markers associated with immature cells of neural lineage, as well as selected markers for neural and glial differentiation. In the present study, we have included markers associated with oligodendrocyte differentiation. The constraints imposed by species specificity continue to limit the individual markers available for work on porcine material.

Two micrograms of total RNA from the sample preparation was reverse transcribed with an Omniscriptase Reverse Transcriptase kit (Qiagen) and 10 µM random primers (Sigma) according to the manufacturer’s instructions. Real-time PCR was performed using a 7500 Fast Real-Time PCR System (Applied Biosystems, Irvine, CA) using Power SYBR Green (Applied Biosystems). Resolution of the product of interest from non-specific product amplification was achieved with melting curve analysis. The gene-specific primers used in this study are shown in Table 1. β-Actin (*ATCB*) was used as an endogenous control to normalize gene expression. The following general real-time PCR protocol was used: denaturation program (95 °C for 10 min), quantification program (95 °C 15 s and 60 °C 1 min) repeated 40 cycles, melting curve program (95 °C 15 s and 60 °C 1 min with continuous fluorescence measurements), and finally a cooling program down to 40 °C. Each reaction was performed in triplicate. Graphs were plotted, and analysis was performed with the ΔΔC_t_ method (7500 Fast System software 1.4 and DataAssist 2.0, Applied Biosystems) and JMP software 4.1 (SAS Americas). All data points are expressed as mean ± standard error (SE). Statistical difference was determined using the Student *t* test. Data were considered significantly different when p<0.05.

**Table 1:** Primers used in quantitative real-time PCR.

**Gene**	**Forward primer (5′-3′)**	**Reverse primer (3′-5′)**	**Product size (bp)**	**Tm °C**
β-actin	acatcaaggagaagctgtgctac	cttcatgatggagttgaaggtagtt	221	60
nestin	ggcttctctcagcatcttgg	aaggctggcataggtgtgtc	150	60
Sox2	acttttgtcggagacggaga	tccgggcagtgtgtacttatc	148	60
vimentin	tcaagtgcctttctgcagttt	tagcacaaggcttcttcggta	143	60
K_i_-67	gaaacccagatccgagcata	cagcagctattctggcaaca	139	60
β3-tubulin	cagagcaagaacagcagctactt	gtgaactccatctcgtccatgccctc	250	60
MAP2	gccatcatacgtactcctcca	agagccacatttggatgtcac	198	60
GFAP	ttgacctgcgacgtggagtc	aggtggcgatctcgatgtcc	225	60
MBP	gagatggctcaactcagaacg	ggttagtatttgccgtgagca	125	60
Claudin 11	atgctcattctgctggctct	gcctgcatacagggagtagc	108	60
KLF4	cagcttcagctatccgatcc	tgccttcaacacaaacttgc	256	60
C-myc	gcccatgaattcacacttgtt	ggatcatgcattcgagaaaaa	123	60
CXCR4	ctgctggctgccatactaca	tccaaggaaagcgtagagga	169	60
JAK1	tttgagaagtccgaggtgcta	caggatctgcttcttcaggtg	147	60
Stat1	agcaagcgtaaccttcagga	gaatctctgggcattttcca	111	60
Stat3	aactcctaggacctggtgtgaa	cgctccctctccttactgataa	193	60

### Immunocytochemistry

Passage-4 pNPCs were dissociated and grown on four-well chamber slides followed by either CNTF or FBS treatment conditions for 24 h. Cells were then fixed for 15 min in 4% paraformaldehyde and then washed three times with PBS, after which a blocking solution consisting of 0.3% Triton X-100 and 5% donkey serum was applied for 1 h, followed by another PBS wash. A panel of antibodies was then incubated overnight at 4 °C to detect antigens expressed by progenitor cells before and after treatment with either CNTF or FBS. These included anti-Sox2 (1:400; Santa Cruz Biotechnology Inc., Santa Cruz CA), anti-K_i_-67 (1:400; BD Biosciences, San Jose, CA), anti-β3-tubulin (1:200; Chemicon), anti-Map2 (1:2,000; Chemicon), anti-glial fibrillary acidic protein (anti-GFAP; 1:400; Chemicon), anti-CXCR4 (1:200; Santa Cruz), and anti-Klf4 (1:800; Chemicon). This was followed by incubation with either antimouse Alexa 546 (1:400; Invitrogen), antigoat Alexa 488 (1:400; Invitrogen), or antirabbit fluorescein isothiocyanate (FITC; 1:800; Chemicon) secondary antibodies. Fluorescence was detected using Leica converse microscopy and Metamorph software (Leica Microsystems Inc., Buffalo Grove, IL). Percentage positive profiles were calculated by counting those profiles expressing specific immunoreactivity in six randomly selected fields, with 4',6-diamidino-2-phenylindole (DAPI) used to determine the total cell number.

## Results

### Morphological changes associated with ciliary neurotrophic factor– and fetal bovine serum–based differentiation conditions

**Figure 1:**
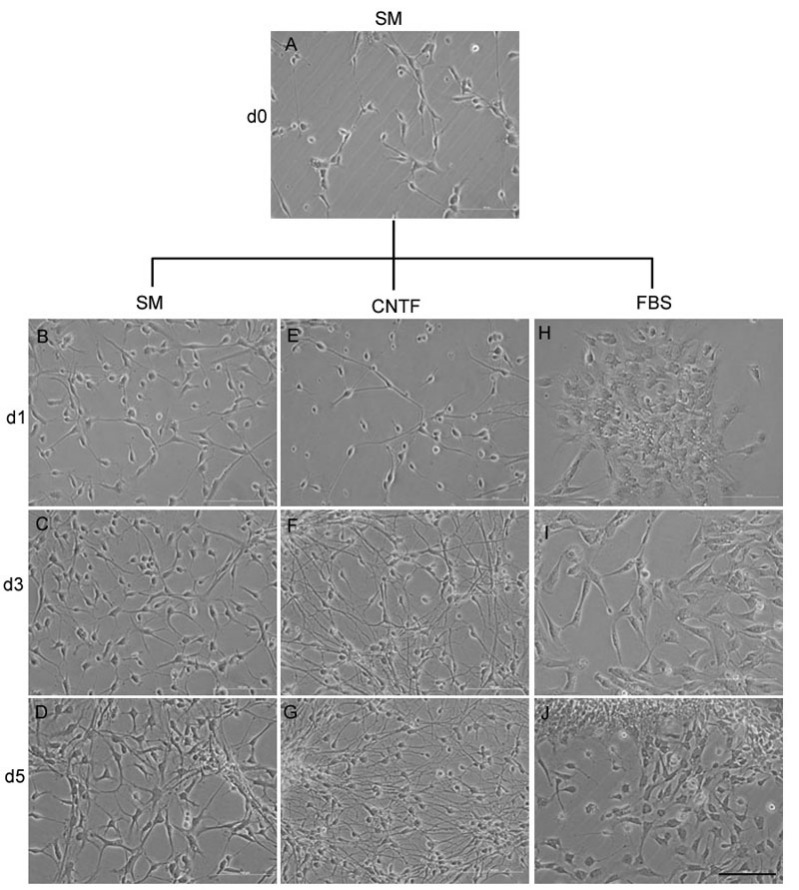
The morphology of porcine neural progenitor cells (NPCs) changes with time in culture and treatment condition. **A**: Phase contrast image of pNPCs just before the initiation of treatment. **B**-**D**: pNPCs maintained under standard proliferation conditions increased in number with time but did not change appreciably in morphology. **E**-**G**: pNPCs cultured under ciliary neurotrophic factor (CNTF) treatment conditions showed an increasing abundance of small cells with long, thin processes. **H**-**J**: pNPCs cultured under fetal bovine serum (FBS) treatment conditions resulted in larger cells with a greater tendency toward polygonal morphology. Scale bar: 100 µm.

Fetal porcine NPC cultures exhibited evidence of cell division on day 1, day 3, and day 5 as the monolayer of cells spread out after plating under standard proliferation conditions (SM), occasionally forming small adherent clusters. Upon cessation of EGF and bFGF, in combination with exposure to either CNTF or FBS, there was morphological evidence of cell differentiation. In CNTF-treated cultures, this consisted of increasing numbers of cells with small somata and long, thin processes, whereas in FBS-treated cultures the cells were larger and exhibited a more polygonal morphology (Figure 1).

### Microarray analysis

**Figure 2:**
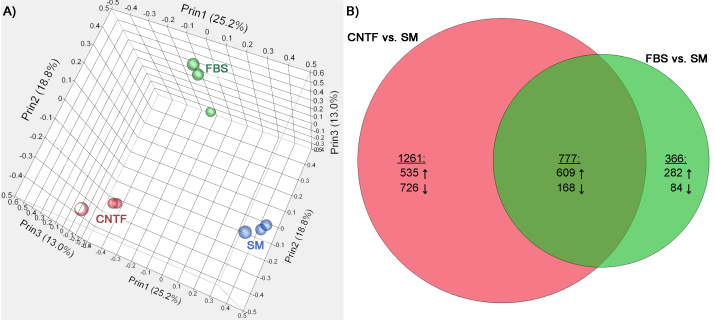
Treatment conditions can be distinguished based on changes in global gene expression using principal component analysis (PCA). **A**: Principal components were calculated for each the three treatments (red=ciliary neurotrophic factor [CNTF), green=fetal bovine serum [FBS], blue=standard medium [SM]). Data from each of these treatments shows considerable separation from the other two, with CNTF and SM at the two extremes. **B**: A Venn Diagram showing significant genes (|fold change|>2, false discovery rate [FDR] α<0.05) unique to either CNTF or FBS treatments (normalized to SM), as well as those genes significantly expressed in both treatments. The top number in each piece is the total gene count in that area, and the numbers below indicate the number of those genes that are upregulated and downregulated.

Three-dimensional principal component analysis revealed clustering of triplicate data sets from each treatment group, together with clear separation between treatment groups (Figure 2A). The first principal component effectively segregated the data in terms of proliferation versus differentiation conditions. The second principal component clearly resolved the differences between the CNTF- and FBS-based differentiation conditions. The third principal component correlated with the variance between replicates within each treatment group. Of the 24,123 genes assayed in the porcine genome, the number with significant fold-change differences (|fold change| > 2; FDR α<0.05) was 2,404. The number of genes upregulated under CNTF-based treatment conditions was 1,144 and the number downregulated 894, out of 2,038 genes that met the significance criteria for this treatment condition (Figure 2B). For the FBS-based treatment, 891 were upregulated and 252 downregulated, out of 1,143 genes that met the significance criteria. The CNTF and FBS treatment groups showed more genes that were upregulated than downregulated, and CNTF treatment showed a higher overall change in gene expression than the FBS group.

### Microarray data from all conditions clustered into four hierarchical groups

**Figure 3:**
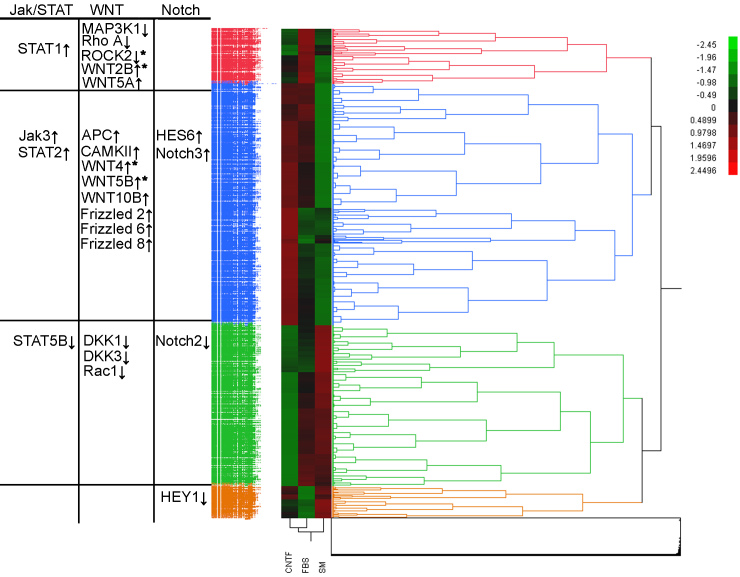
Heat map analysis revealed that the 5,666 genes exhibiting statistically significant changes in expression level (false discovery rate [FDR] α<0.05) could be clustered into one of four groups. Genes from the Jak/STAT, Wnt, and Notch pathways are shown relative to positioning in the relevant clusters, along with arrows next to each gene name indicating if the gene is upregulated or downregulated. Asterisk (*) indicates significant fold change (|fold change|>2).

A heat map and a hierarchical cluster diagram were generated from all genes with statistically significant changes in expression level, regardless of the magnitude of fold-change. Data from the 5,666 genes that met an FDR correction of α<0.05 were analyzed to reveal correlations in expression behavior between genes and treatment groups (Figure 3). From this analysis, four clusters emerged, containing 2,794, 1,864, 635, and 373 genes, respectively. DAVID analysis was then performed on these four clusters (Appendix 1). One cluster (shown in blue in Figure 3) contained major histocompatibility complex (MHC) class II genes, as well as genes related to transcription, protein processing, immune response, and three groups related to synaptic transmission. A separate cluster (shown in green) contained genes related to cell cycle and mitosis, several metabolic and biosynthesis pathways, as well as genes involved in angiogenesis and blood vessel formation. A third cluster (red) contained MHC class I genes and genes related to immunity. The smallest cluster (orange) showed only two significant categories from the DAVID analysis, namely, small molecule transport and nicotinamide adenine dinucleotide–related metabolism.

The potential involvement of selected signaling pathways, namely, Jak/STAT, Notch, and wingless-type (Wnt), was also investigated regarding the hierarchical clustering observed. The largest cluster, associated with differentiation, showed upregulation of genes in all pathways examined, with genes in the Wnt/Frizzled pathway particularly well represented. In the cluster associated with immature cells, genes were downregulated across the same pathways. Results from the smaller, FBS-related clusters were more mixed.

### Comparison of treatment-induced gene expression changes using volcano plots

**Figure 4:**
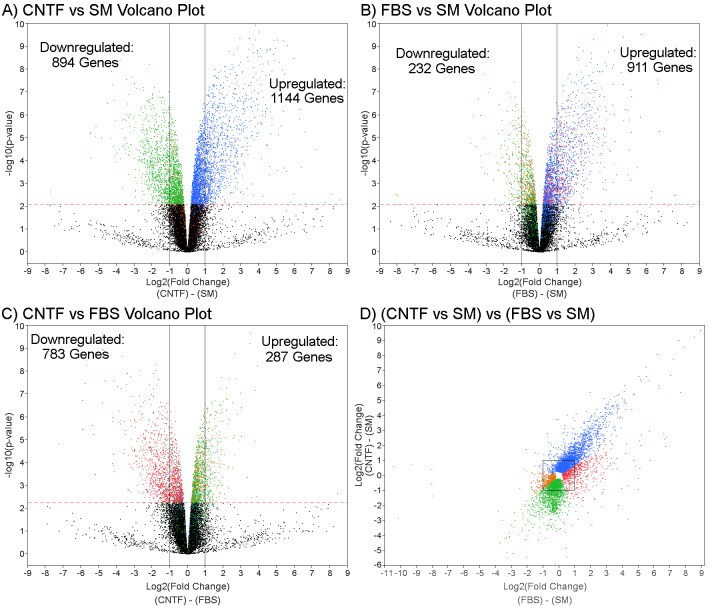
The use of volcano plots allows for comparison between conditions. Volcano plot displays of genes significantly upregulated and downregulated as a result of different treatments: **A**: Ciliary neurotrophic factor (CNTF)-based conditions using standard medium (SM) as baseline, (**B**) fetal bovine serum (FBS)-based conditions using SM as baseline, and (**C**) CNTF using FBS as baseline (**C**). With an false discovery rate (FDR) multiple test correction of α<0.05, the p-value cutoff (indicated by red dashed line) was p<.00794424 in (**A**) and (**B**), and was p<0.00554170 in (**C**). The lines drawn at −1 and 1 show the log2 cutoff for significant fold change in **A**-**C**. **D**: Comparison of significantly changed genes for CNTF and FBS treatments, using SM as a control in both cases. The box in (**D**) is the log2 cutoff for fold change significance. The colors of the data points correspond to the hierarchical cluster analysis in Figure 3 and therefore illustrate the direction of expression changes for genes in those clusters, relative to treatment condition.

Volcano plots were used to further evaluate global gene expression between treatment groups, based on the magnitude and significance of the changes induced (Figure 4). Comparison of CNTF-based treatment to SM showed that more genes were upregulated than downregulated (Figure 4A). Comparison of FBS-based treatment to SM showed an even greater proportion of upregulated as opposed to downregulated genes (Figure 4B). Comparison of CNTF- versus FBS-based treatments showed that a higher number of genes were downregulated following CNTF- compared to FBS-based treatment (Figure 4C). Another comparison of CNTF- versus FBS-based treatment conditions was performed in which fold changes were compared directly. This comparison also showed a higher number of genes were upregulated in the FBS-based treatment, compared to CNTF-based treatment, and a higher number were downregulated in the CNTF-based treatment compared to FBS-based treatment (Figure 4D). Most genes that were upregulated in the CNTF-based treatment were also upregulated in the FBS-based treatment (Figure 4D).

### Identification of genes exhibiting significant changes in expression level

After the behavior of gene expression levels overall was examined, specific genes were identified, and how their expression was influenced by the differentiation conditions employed was considered. Among the known genes in the pig genome, those relating to signal transduction pathways, gene transcription, and cell cycle control were selected for individual inspection (Appendix 2). The genes listed met our chosen significance criteria (|fold change| >2, FDR α<0.05) for the CNTF-based treatment condition, and were then compared further. Most of the genes listed related to the cell cycle were downregulated with differentiation, whereas those related to cell signaling, particularly of the Wnt pathway, were upregulated. Markers for the three mature neural progenitor lineages, namely, neurons (doublecortin [*DCX*], tubulin, beta 3 [*TUBB3*], neurofilament light polypeptide [*NEFL*]), astrocytes (aquaporin 4 [*AQP4*], glial fibrillary acidic protein [*GFAP*]), and oligodendrocytes (myelin basic protein [*MBP*]) were upregulated (Appendix 2). Genes related to the development of neural circuitry were also upregulated, including axonal projections (ephrin type-A receptor 4 [*EPHA4*], roundabout homolog 1 [*ROBO1*]) and synaptic function (synaptic vesicle 2-related protein [*SVOP*], synaptoporin [*SYNPR*], synaptotagmin-1 [*SYT1*]; Appendix 2). Certain immune-related genes were also upregulated, including several MHC class II antigens and one class I antigen, interleukins and IL receptors (*IL-7R*, *IL-13Ra*, and *IL-16*), several other surface molecules (cluster differentiation [*CD14*], *CD74*, *CD180*), and Toll-like receptor 2 (*TLR2*). Each gene symbol is colored based on its location in the hierarchical clusters in Figure 3, of which most upregulated genes correlate to the blue cluster, and most downregulated correlate to the green cluster.

### Confirmation of clustering by treatment condition using quantitative real-time polymerase chain reaction

**Figure 5:**
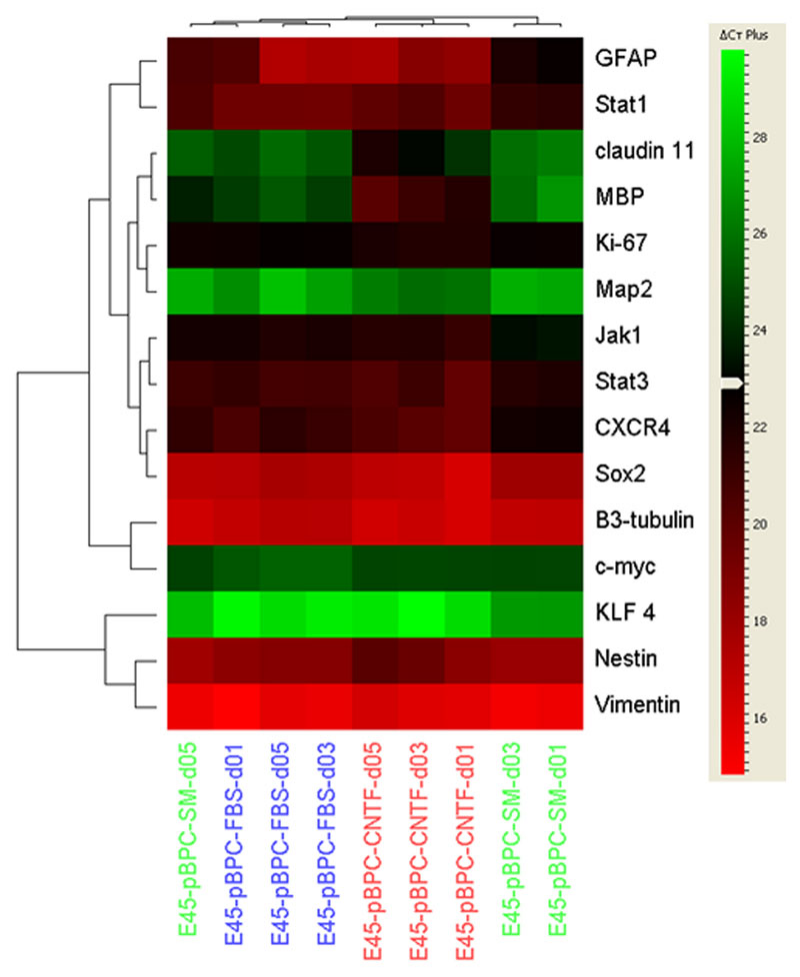
Expression patterns of selected genes were confirmed using quantitative real-time polymerase chain reaction (PCR). Confirmation of selected immature and lineage-related genes was undertaken using quantitative real-time PCR and displayed as a global view heat map across treatment conditions and time points in culture, the latter indicated along the abscissa. As indicated by the group labels, all porcine neural progenitor cultures were originally derived from brain tissue at embryonic day 45 (E45-pBPC). Untreated controls (standard medium [SM]) have titles in green text, ciliary neurotrophic factor (CNTF) treatment in red, and fetal bovine serum (FBS) in blue. The results were further subjected to cluster analysis employing Pearson’s correlation coefficient as a distance measure, as indicated by dendrograms at top and left. For the heat map scale (right), black is set as the median of all ΔCT values (23) obtained in the study, while increasing intensity of red represents higher gene expression levels and increasing green shows lower levels.

In addition to the microarray results, selected immature and lineage-related genes were analyzed using quantitative real-time PCR. The results were further subjected to cluster analysis employing Pearson’s correlation coefficient as a distance measure (Figure 5). Results showed an evident trend toward clustering by treatment group, with only one of the nine groups countering the trend. On closer examination, that particular case involved the untreated (SM) group at day 5 associating with the FBS group at day 1, perhaps reflecting a degree of spontaneous differentiation in the former vis-à-vis relatively modest treatment-induced changes in the latter. Apart from those two cases, there was also a trend for clusters to order according to time point (treatment day). In terms of individual gene categories, there was clustering of the progenitor markers nestin, vimentin, and Krüppel-like factor 4 (*KLF4*) across treatments and time points, as there was for the oligodentrocyte markers myelin basic protein (*MBP)* and claudin 11. Pathway genes *Jak1* and *Stat3* associated most closely with *Sox2* and *CXCR4*, while *STAT1* clustered with the astrocyte-associated marker *GFAP*.

### Changes across a gene expression profile with quantitative real-time polymerase chain reaction

**Figure 6:**
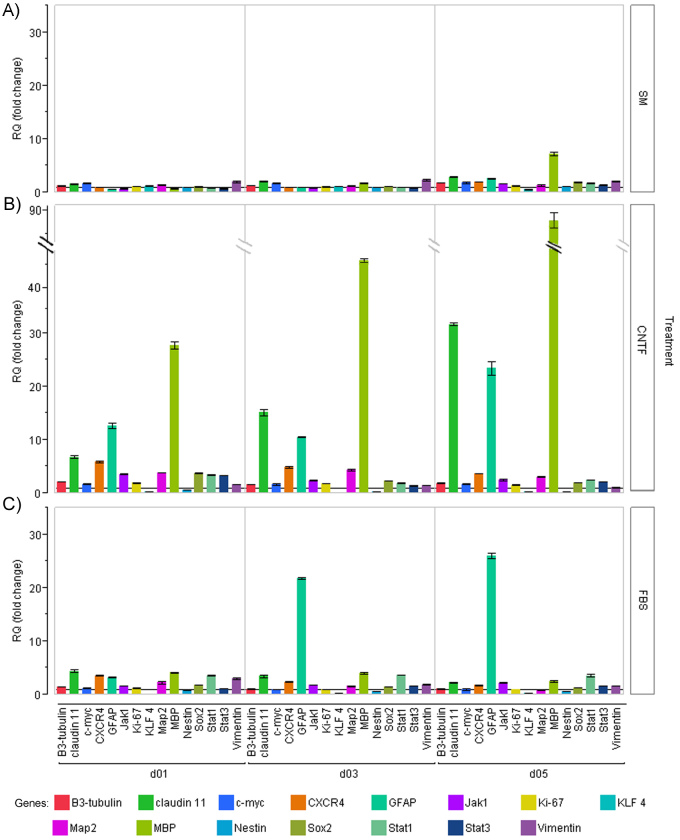
Genes were examined during the time course of treatment using real-time polymerase chain reaction (PCR). Fifteen genes were profiled across five days following treatment with either standard medium (SM, **A**), ciliary neurotrophic factor (CNTF; **B**), or fetal bovine serum (FBS; **C**). Fold changes in expression level were calculated with RQ using the ΔΔCt method, normalized to SM day 0. Samples were run in triplicate and both CNTF and FBS conditions were compared to the respective SM data for days 1, 3 and 5, with significance established using the Student’s *t*-test. Error bars represent standard error of the mean (SEM). *=p<0.05.

The expression of 15 transcripts was determined with quantitative real-time PCR, and the results obtained from cells cultured under proliferation conditions were compared to those from cells cultured under differentiation conditions. After normalization to the expression levels of untreated controls at day 0, differential expression was compared for cells undergoing the CNTF- and FBS-based treatments and compared to the cells maintained in SM over the identical period. Comparison of overall expression changes across all genes analyzed showed modest variability in gene expression for the SM-treated group, with the exception of higher MBP expression at day 5 (Figure 6A). Interestingly, this could have contributed to the clustering anomaly noted in Figure 5, especially taken together with the relative lack of GFAP induction at day 1 for the FBS group. Looking at treatment condition, several genes showed very large fold changes across time following CNTF-based treatment, as claudin 11, *MBP*, and *GFAP* all trended to higher expression levels with time (Figure 6B). FBS-treated cells showed higher *GFAP* expression, particularly beyond day 1, with comparatively restrained changes in other genes (Figure 6C).

### Quantitative changes in porcine neural progenitor cell transcripts under differentiation conditions

**Figure 7:**
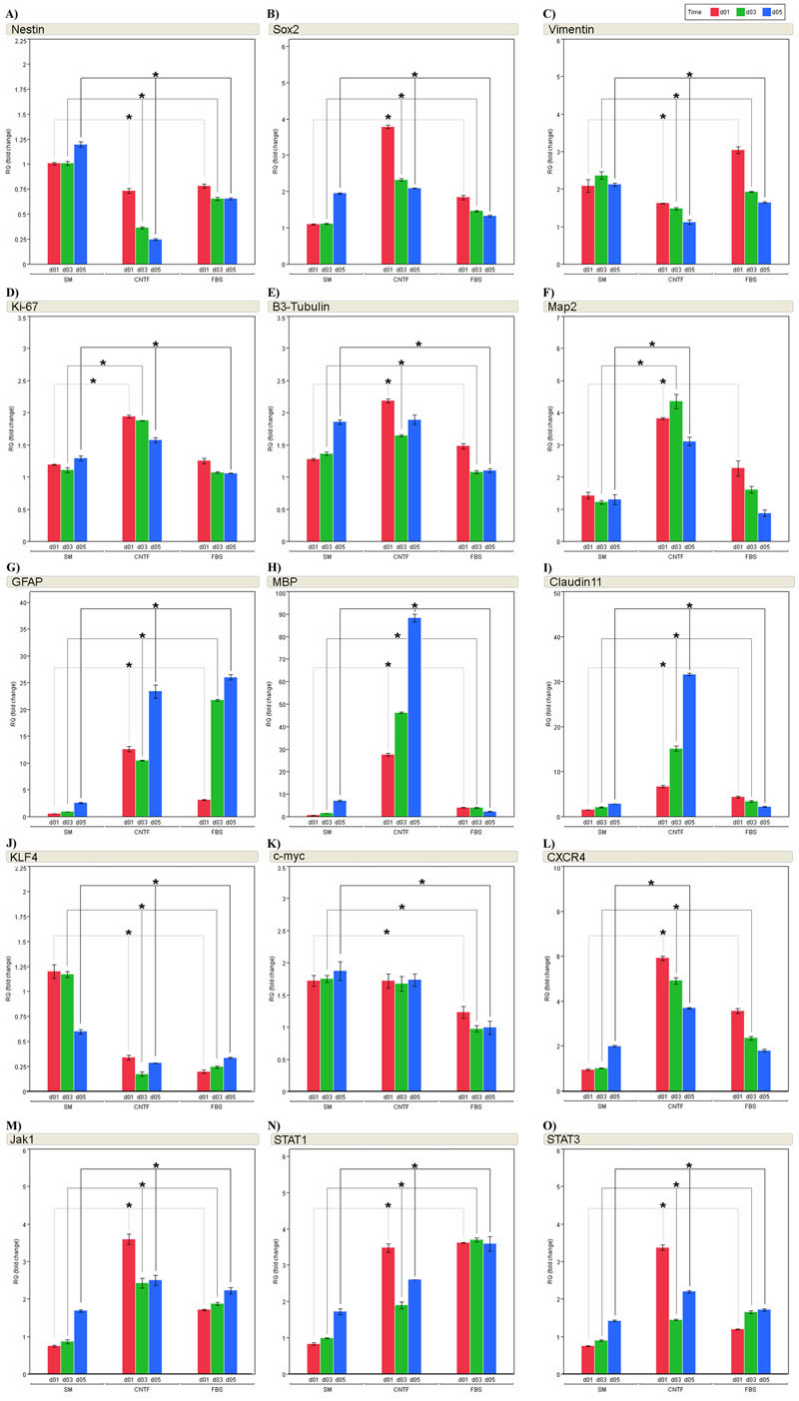
Examination of 15 selected gene expression profiles using polymerase chain reaction (PCR) shows significant changes with treatment and time. Progenitor markers nestin (**A**), Sox2 (**B**), Vimentin (**C**), K_i_-67 (**D**), Krüppel-like factor 4 (Klf4; **J**), c-myc (**K**), and C-X-C chemokine receptor type 4 (CXCR4; **L**) are profiled across time for each treatment. Mature neural markers β3-tubulin (**E**) and Map2 (**F**) as well as mature glial markers glial fibrillary acidic protein (GFAP; **G**), myelin basic protein (MBP; **H**), and Claudin 11 (**I**) are profiled as well. Changes to gene expression relating to the Jak-STAT signaling pathway, Janus kinase 1 (Jak1; **M**), signal transducer and activator of transcription 3 (STAT1; **N**) and signal transducer and activator of transcription 3 (STAT3; **O**), are also shown. Fold changes for the 15 gene profiles were normalized to standard medium (SM) day 0. Data from treatment day 1 are shown as red bars, day 3 as green bars, and day 5 as blue bars. Both fetal bovine serum (FBS) and ciliary neurotrophic factor (CNTF) treated groups are compared to SM, respective to time point, and then significance was established using the Student’s *t*-test. Error bars represent standard error of the mean (SEM). *=p<0.05. β-Actin was used as the endogenous control.

The profile of 15 markers was then examined by individual gene for each treatment condition and treatment day. The progenitor markers included nestin, *Sox2*, and vimentin, together with the proliferation marker *K_i_-67* (Figure 7A-D). Significant downregulation of nestin expression was observed for the CNTF (up to approximately fourfold, transcript levels: d1, 0.74; d3, 0.37; d5, 0.25), and FBS (approximately 1.6 fold, transcript levels: d1, 0.79; d3, 0.66; d5, 0.66) treatment conditions, compared to matched controls cultured under standard proliferation conditions (Figure 7A). In contrast, *Sox2* was upregulated in response to either CNTF or FBS treatment (Figure 7B), although more obviously for the CNTF group. Of note, in each case the response was most prominent at day 1 of treatment and rapidly diminished on subsequent days. Vimentin was generally upregulated across the control and treatment groups compared to the baseline controls from day 0, indicating a time-related shift toward greater vimentin expression during the culture period. Compared to the time-matched untreated controls, however, vimentin was generally downregulated in the CNTF-based and FBS-based treatment groups, although day 1 in FBS was transiently higher (Figure 7C). *K_i_-67* showed modest upregulation in the CNTF-based treatment condition, whereas this was not observed for the FBS condition (Figure 7D). In both cases, *K_i_-67* expression appeared to trend downward with time.

Samples from the same treatment groups were also analyzed for the expression of five differentiation markers (β3-tubulin, *Map2*, *MBP*, claudin 11, *GFAP*). These data revealed evidence of mixed neuronal and glial differentiation associated with both treatment populations (Figure 7E-I). The neural marker β3-tubulin was only modestly altered, being slightly upregulated (1.1–2.2 fold) in the CNTF and FBS groups (Figure 7E). In contrast, there were notable (>threefold) increases in message for the mature neuronal marker *MAP2* in response to the CNTF condition across time points together with a substantial, but lower, response in the FBS condition that returned to control levels at the latest time point (Figure 7F). Two oligodendrocyte markers, *MBP* and claudin 11, were among the two most dramatically altered transcripts in the study, with *MBP* upregulated more than 88 fold in the CNTF condition at day 5, along with claudin 11, which increased 32 fold. Both transcripts increased only two- to fourfold in the FBS condition (Figure 7H,I). The glial marker *GFAP* was also notably upregulated, increasing more than 23 fold in the CNTF condition and greater than 26 fold in the FBS condition by the final time point (Figure 7G).

Two nuclear transcription factors related to proliferation were also examined. Expression of *KLF4*, a member of the Krüppel-like family of transcription factors, was strongly decreased up to fivefold (transcript level approximately 0.2) in the CNTF and FBS treatment conditions (Figure 7J). Of the transcripts tested, *KLF4* was the most strongly downregulated in response to differentiation conditions. Transcripts for *c-Myc* were also downregulated, but only in the FBS condition (Figure 7K).

In addition, transcripts levels for the chemokine receptor *CXCR4*, which is associated with cell homing behavior, were strongly increased (up to 5.9 fold) in the CNTF- and FBS-based treatment conditions (Figure 7L). These elevated levels peaked on day 1 and exhibited a clear downward trend over the remainder of the treatment period. Finally, the signaling pathway genes *Jak1*, *STAT1*, and *STAT3* were also examined, and they were upregulated in the CNTF- and FBS-based treatment conditions to varying degrees (Figure 7M-O).

### Validation of the microarray data via quantitative real-time polymerase chain reaction

The results of the quantitative real-time PCR analysis were used to validate the microarray data (Table 2). For the CNTF-based treatment group, the data sets showed similar trends. Examined in detail, however, the microarray data for β3-tubulin, *CXCR4*, *GFAP*, and *Jak1* exhibited variance regarding the quantitative real-time PCR results across treatment groups.

**Table 2:** Comparison of microarray data and real-time PCR data.

	**CNTF(-SM)**			**FBS(-SM)**				**CNTF(-FBS)**				
**Gene**	**Microarray**	**qPCR**	**Confirmed?**	**Microarray**		**qPCR**	**Confirmed?**	**Microarray**	**qPCR**	**Confirmed?**		
**Immature Markers:**												
Nestin	−4.85*	−2.58*	✓	−1.37		−1.53*	✓	−3.54*	−1.68*		✓	
SRY (sex determining region Y)-box 2 (Sox2)		2.12*				1.17*			1.82*			
Vimentin	−1.19	−1.51*	✓	−1.02		1.01*	?	1.04	−1.52*		?	
c-myc	−1.61	−1.03	X	−1.37		−1.69*	✓	−1.18	1.63*		X	
Kruppel-like factor 4 (KLF4)	−3.42*	−3.63*	✓	−1.33		−3.86*	✓	−2.57*	1.06		X	
K_i_-67	1.09	1.49*	✓	1.08		−1.05*	X	1.05	1.56*		✓	
C-X-C chemokine receptor type 4 (CXCR4)	1.05	3.52*	✓	−1.02		1.93	?	1.07	1.82*		✓	
**Differentiation Markers:**												
Janus kinase 1 (Jak1)	1.33	2.56*	✓	−1.31	1.75*		X	−1.15	1.46*			X
STAT1	1.35	2.24*	✓	2.32*	3.06*		✓	−1.72	−1.37*			✓
STAT3	1.22	2.28*	✓	1.12	1.48*		✓	1.02	1.54*			?
β-3 Tubulin	2.08*	1.27	X	2.07*	−1.27*		X	1	1.56*			?
Microtubule-associated protein 2 (Map2)	1.47	2.84*	✓	1.16	1.2		✓	1.27	1.33*			✓
Glial fibrillary acidic protein (GFAP)	5.05*	10.87*	✓	7.13*	11.90*		✓	−1.41	−1.10*			✓
Myelin basic protein (MBP)	8.81*	16.84*	✓	1.26	1.09*		✓	6.96*	15.41*			✓
Claudin 11 (CLDN11)	16.33*	7.92*	✓	1.43	1.52		✓	11.44*	5.22*			✓

Microarray and real-time PCR data from CNTF treated cells were normalized to SM treated cells; data from FBS treated cells were normalized to SM, and data from CNTF treated cells were normalized to FBS. Noted differentiation and proliferation marker genes listed were compared in both the microarray and real-time PCR data, with blank cells in the microarray signifying no known gene annotation in the Affymetrix probe list. The real-time PCR data are from the day 5 time point only. * indicates significant gene (microarray: |fold change| > 2, FDR α<0.05; real-time PCR: p<0.05, fold change ≠ 1) **✓** indicates similar fold change direction between microarray and real-time PCR **X** indicates differing direction of fold change between microarray and real-time PCR or lack of PCR significance **?** indicates no clear correlation between the fold change of microarray and real-time PCR.

### Protein expression examined using immunocytochemistry

**Figure 8:**
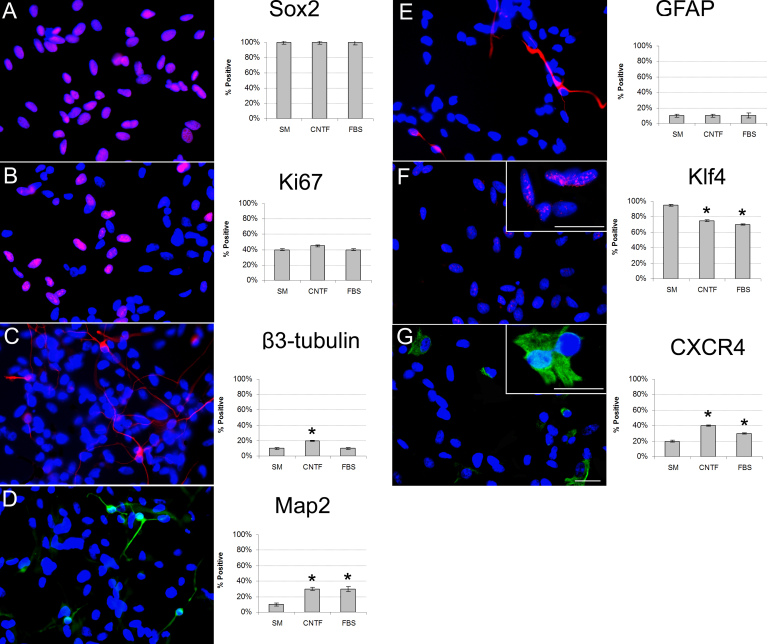
Changes in protein expression were identified using immunocytochemical analysis. Expression of seven selected markers were confirmed as proteins. Markers examined included Sox2 (**A**), K_i_-67 (**B**), β3-tubulin (**C**), Map2 (**D**), glial markers glial fibrillary acidic protein (GFAP; **E**), Krüppel-like factor 4 (KLF4; **F**), and C-X-C chemokine receptor type 4 (CXCR4; **G**). Insets provide higher power images of labeled profiles to illustrate labeling patterns. Photomicrographs showing labeling for each marker under standard medium (SM), ciliary neurotrophic factor (CNTF), and fetal bovine serum (FBS) conditions from left to right, respectively. Insets in **F** and **G** provide higher power images of labeled profiles, to illustrate labeling patterns. In addition, results after 24 h treatment were compared across treatment conditions as shown in the accompanying histograms. In each case, untreated cultures in SM are represented by the left bar, the CNTF-based condition by the center bar, and the FBS-based condition by the right bar. Error bars show standard deviation (SD); asterisks (*) denote statistical significance (p<0.05). Scale bar: 100 µm.

**Figure 9:**
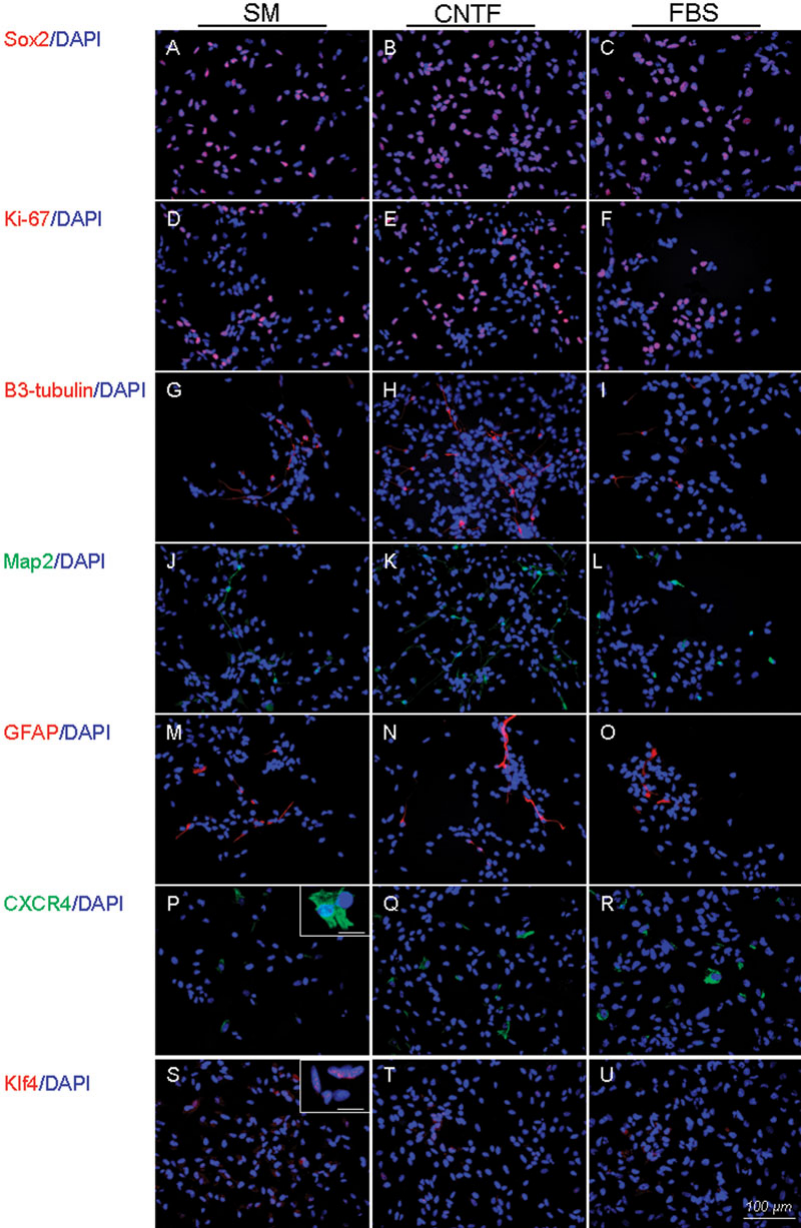
Changes in selected marker proteins showing changes in relative expression levels for each treatment condition. This figure expands on the immunocytochemical data presented in Figure 8 by showing the results obtained for the same seven markers under baseline proliferation (standard medium [SM]) conditions (**A**, **D**, **G**, **J**, **M**, **P**, **S**), as previously shown, together with the ciliary neurotrophic factor (CNTF; **B, E**, **H**, **K**, **N**, **O**, **T**) and ftela bovine serum (FBS; **C**, **F**, **I**, **L**, **O**, **R**, **U**) treatment conditions for comparison. Markers are listed to the left of the data, treatment above. Scale bar: 100 µm.

Immunocytochemistry was used to confirm marker changes at the level of protein expression for the CNTF and FBS treatment conditions, as well as to examine the distribution of labeling for specific markers within the cultured cell population. After 24 h, the immature markers Sox2, K_i_-67 (Figure 8A,B), and Klf4 (Figure 8F), the lineage markers β3-tubulin, Map2, and GFAP (Figure 8C,D,E), and the surface marker CXCR4 (Figure 8G) were evaluated in treated cultures and untreated controls (see also Figure 9). To do this, the percentage of immunopositive cells was calculated for each marker under each condition. Of the immature markers, KLF4 was most closely associated with decreased expression, consistent with loss of progenitor status. Of the neuronal markers, Map2 was equally upregulated under both differentiation conditions, whereas β3-tubulin appeared to be increased over baseline only in the CNTF condition. The glial marker GFAP was expressed by a similarly sized subpopulation of cells across conditions, although the labeling intensity was more prominent in the differentiating cultures, suggesting increased expression levels. CXCR4 labeling also increased in differentiating cultures, more so in the CNTF condition.

## Discussion

The pig has recently emerged as a valuable large animal model for use in experimental medicine, particularly in situations where rodent models do not adequately represent the challenges faced when translating new interventions to the clinical setting. Stem cell technology is one field in which results in rodents often need to be reproduced on a larger scale, using techniques suitable for human application. Due to immunological considerations, the essential starting point for work in stem cell transplantation is deriving and characterizing allogeneic cells. Despite the obstacles presented by working with large animals, there has been recent progress culturing stem cell populations from porcine tissue, including proliferation-competent progenitors from the brain [31,33,41] and neural retina [32,33]. Although molecular analysis of these porcine CNS progenitors has been somewhat constrained by a lack of species-specific probes, ongoing sequencing of the *Sus scrofa* genome now permits the design of porcine microarrays as well as pig-specific primers for use in quantitative real-time PCR analysis. The present study takes advantage of these new tools and shows for the first time the magnitude and temporal characteristics of the transcriptional response of porcine forebrain progenitors following exposure to differentiation conditions in culture. Several changes seen have implications for the tolerance and integration of grafted pNPCs.

In this study, two alternate conditions were tested, both commonly used for in vitro determination of NPC multipotency. The methods differ with one being defined in terms of chemical composition (CNTF) and the other (FBS) undefined. Both were associated with changes in gene expression consistent with the induction of differentiation in pNPCs, including the downregulation of immature markers and the upregulation of lineage markers, thereby confirming previous studies reporting in vitro differentiation of these cells. In addition, this study confirms at the transcriptional and protein levels the upregulation of markers reflecting neuronal and glial subpopulations within the differentiating cultures, as previously indicated by immunolabeling alone [31,32] and extends those findings by delineating the relative magnitude and temporal characteristics of the changes in a manner that allows comparison between treatment conditions. From this it was evident that the overall pattern of change seen across the transcription profile was largely similar between treatment groups and that the major difference was magnitude of effect. Specifically, although both treatments resulted in elevated markers for neuronal and glial lineage, the CNTF-based treatment condition showed quantitatively greater expression of the neuron- and glial-associated markers MBP, claudin 11, and Map2, together with greater induction of the neural migration-associated marker CXCR4, whereas the FBS-based treatment condition resulted in greater induction of the astrocyte-associated marker GFAP. These findings support the concept that use of defined, serum-free differentiation conditions confers advantages over serum in terms of promoting the relative yield of cells with neuronal phenotype.

In previous work, we investigated the transcriptome of porcine NPCs using a human microarray [31]. In the current study, we present the results of what is, to our knowledge, the first investigation of brain-derived pNPCs using porcine-specific microarrays, as well as the first examination of global changes in the pNPC transcriptome during differentiation. The results obtained reveal the identity and relative proportion of transcripts being upregulated and downregulated over the course of in vitro differentiation, together with a comparison between non-defined (FBS) and defined (CNTF) conditions. These results confirm the differentiating properties of both conditions, while also confirming that the CNTF condition is the more favorable of the two for relative yield of neuronal phenotype.

The data also bring to attention several additional interesting features and novel findings for the changes observed. Given the evidence in favor of the differentiating qualities of the CNTF condition, the involvement of the Jak/STAT, Wnt, and Notch pathways, as illustrated in the hierarchical cluster analysis, is consistent with cellular differentiation. For instance, the Jak/STAT pathway has been shown to initiate astrogliogenesis [42] in a CNTF-dependent manner [43] and, through STAT5, to induce proliferation of NPCs [44], consistent with the results obtained here. *Wnt 4* and *Wnt 5B* have been previously implicated in NPC differentiation [45]. Wnt 10B has been implicated in differentiating epithelial stem cells in the skin [46] and might also play a role in differentiating NPCs, as this *Wnt* gene was also significantly upregulated in the present study. Dkk-1, an inhibitor of the Wnt pathway, has been implicated in differentiating ES cells into NPCs [47]. In terms of the Notch pathway, Notch 2 and 3 have been shown to be upregulated in the developing brain [48]. Hes6 has been shown to inhibit astrocyte formation and favor neurogenesis in NPCs [49]. Hey1 aids in maintaining NPCs in the developing brain [50], and here was more altered by the FBS treatment condition.

Differentiation is a predominant theme evident in the gene expression changes seen under the conditions employed here, and several markers associated with immune function also show significant upregulation, particularly in response to CNTF-based treatment. Of these, among the most prominent are the MHC class II antigens (Appendix 2). There have been prior reports of upregulation of these antigens by differentiating cells [51]. MHC class II upregulation has been shown to be induced by related ligands such as LIF and by activation of the Jak/STAT pathway, particularly through upregulation of the STAT1 gene [52]. Importantly, any changes in MHC class II expression represent an important consideration in the context of NPC transplantation and graft tolerance. Other immune-related genes upregulated following CNTF treatment include several interleukins, specifically interleukin 7 receptor (IL-7R) and interleukin 16 (IL-16). Interleukin 7 may play a trophic role during differentiation in the developing brain [53], for instance, in glial development [54]. Interleukin 16 may also have a role in the developing brain, particularly in microglial activation [55].

Other upregulated genes with putative relationships to neurogenesis include TLR2 and bone morphogenetic protein 2. Bone morphogenetic protein 2 has been reported to induce neurogenesis in NPCs [56], and TLR2 has been implicated in neurogenesis in the adult murine hippocampus [57]. Both signaling-related molecules may relate to the preferential generation of neurons seen in the CNTF-based treatment condition.

A different aspect of the data that merits consideration is the dynamic pattern of changes seen in transcript expression. One important observation from the quantitative real-time PCR data are that the expression of some transcripts varied in control pNPCs maintained under standard proliferation conditions for the five-day course of the experiment. This finding reveals variability in gene expression that is not a direct function of treatment condition and points out the value of using time-matched as well as baseline controls in studies of this type. This finding also implies that changes up to approximately twofold should be interpreted with caution for any given gene since this might reflect factors unrelated to treatment condition.

Another interesting feature of the temporal pattern relates to whether transcript expression level amplifies with time or peaks early and then trends back toward control levels. In the present study, an example of the first pattern is represented by GFAP, which trended upward with duration of treatment. In addition, nestin trended downward for the CNTF condition. In contrast, an example of an abrupt change in transcript expression that subsequently decelerated back in the direction of control levels is represented by CXCR4, together with Sox2 for CNTF and MAP2 for FBS. The former, amplifying pattern would appear to reflect transcriptional changes of a more sustained nature as might be anticipated for marker changes associated with terminal differentiation. The latter, more transient, pattern might reflect short-term reactive responses to altered culture conditions. The transient quality of CXCR4 expression, a receptor involved in stem cell homing, is of particular interest in that it has potential implications for the timing of transplantation and successful integration of grafted cells.

Considering specific markers, the present data support the use of nestin as a marker of relative immaturity for porcine NPCs, as shown in other species, specifically in preference to the other neural progenitor markers vimentin and Sox2. Likewise, the lineage markers MBP, claudin 11, MAP2, and GFAP were notably responsive to treatment conditions, as anticipated from prior work. Also examined here were two markers less frequently monitored, namely, KLF4 and CXCR4, and expression of these genes exhibited prominent treatment-associated effects at the RNA and protein levels. KLF4 is a zinc finger transcription factor involved in diverse cell functions, including proliferation [58], differentiation, and apoptosis [59,60]. Recently, KLF4 has received considerable attention as one out of a set of four transcription factors whose combined overexpression is sufficient to reprogram mouse and human fibroblasts into induced pluripotent stem cells [61,62]. Since KLF4 overexpression can obviously contribute to increased phenotypic plasticity, it is perhaps not surprising that the induction of differentiation, which involves phenotypic restriction, would be accompanied by decreased transcription of this gene. What was particularly striking is that KLF4 decreased more abruptly at the transcript and protein levels than did other markers of immaturity. Therefore, knowing whether diminished KLF4 expression represents a novel and sensitive indicator of the loss of progenitor status during the differentiation of NPCs would be interesting.

In contrast, expression of the CXC chemokine receptor 4 (CXCR4, fusin, CD184) was rapidly and strongly elevated in response to the CNTF-based and FBS-based treatment conditions. Multiple cell types in the mammalian brain have been shown to possess chemokine receptors [63], including the expression of CCR3, CXCR4, CXCR2, and CX3CR1 on neurons, CXCR4 on astrocytes, and CCR3 and CCR5 on microglia. Stromal cell-derived factor-1 (SDF-1) is known to serve as a ligand for CXCR4, and this signaling pathway has a documented role in mediating cell migration, precursor cell proliferation, and neuronal circuit formation during neural development and is possibly involved in regulating cell migration in response to injury [34,64–67]. In previous work, we reported expression of SDF-1, and tentatively CXCR4, by porcine NPCs [31] as well as expression of CXCR4 by feline NPCs [68]. In those instances, the NPCs were maintained under proliferation conditions. Here we extend those findings by demonstrating significant upregulation of CXCR4 expression under CNTF and FBS differentiation conditions, suggesting a role for this surface receptor during the transition from multipotent neural progenitor to committed precursor. This developmental time window also corresponds to the period of active cell migration during neurogenesis in the mammalian CNS. The transient quality of CXCR4 expression during differentiation has potential implications for the timing of transplantation and successful integration of grafted cells.

Previous differentiation studies involving rat and mouse retinal progenitor cells (RPCs) presented results comparable in aspects to our current pig study. Rat RPCs were studied by Bhattacharya et al. [24] who looked at pathways involved in differentiation, particularly the Jak/STAT, MAPK, and Notch pathways. The researchers found an increase in the protein levels of Notch 1 and Hes5 following CNTF treatment, while our porcine brain cells did not show an increase in the respective mRNA levels of those particular genes. The mouse RPC study by Rhee et al. [21] included a microarray analysis of gene transcript levels following CNTF treatment. Several of the gene expression changes Rhee et al. list are mirrored in the present study, although here the changes did not reach our significance criteria, such as synapsin II, nucleolin, annexin A7, ephrin-B2, STAT1, and STAT3.

The present study contributes to the small but growing literature on porcine NPC differentiation and confirms some previous findings [36,69] while also introducing several novel observations. Differences between studies might reflect methodological differences including the details of the treatment conditions used, as well as the preferential examination of transcript expression, as opposed to proteins. In a proteomics study, Skalnikova et al. [36] reported gene expression changes for α-centractin, α-B crystalline, and mitochondrial medium-chain specific acyl-CoA dehydrogenase that were similar in direction to what we observed with microarray, although they did not meet our significance criteria. Some of the genes Skalnikova et al. reported as being upregulated, including heat-shock protein β-1 and hnRNP H, were not corroborated by our transcript-based data. In terms of pathway analysis, we found several genes with fold changes similar to their findings, including Alk, cJun, CaMKIIα, ERK5, JNK, and CASP1, although only CASP1 reached significance here. At least some of these genes may play a role in non-specific responses to stress, and therefore the data may in part reflect differences in laboratory protocols.

In general terms, the present study provides additional evidence of the extent to which molecular findings related to neural progenitor cell behavior can be extended from rodents to a large animal model, in this case the pig. Such models have importance in translational research, particular for surgical approaches at the organ level. The present results also reinforce our previous findings for murine retinal progenitors and reveal a similar pattern of differentiation as a function of CNTF-based, versus serum-based, treatment. Together, these results are consistent with a broad conservation in differentiation characteristics between CNS progenitor populations across mammalian species, a notion that has been often been assumed but only recently examined more systematically. Significantly, the present work also reveals short-term dynamics in transcript levels, relevant to timing of phenotypic assays and suggestive of complexities in the regulatory process occurring during NPC differentiation worthy of further investigation. In particular, the abrupt and substantial changes seen in KLF4 and CXCR4 expression at the transcript and protein levels suggest that regulation of these genes is sensitive to changes occurring during the transition of NPCs from multipotent progenitors to specified post-mitotic precursors. Furthermore, upregulation of MHC genes can result from various culture conditions, including differentiation treatments, and this should be considered when interpreting the results of transplantation studies, particularly regarding graft tolerance.

## Appendix 1. Classes of genes expressed in each hierarchical cluster.

Genes from the cluster analysis shown in Figure 3 were subjected to DAVID to determine the types of genes expressed in each of the four hierarchical clusters. Entries shown were selected when p<0.05. To access the data, click or select the words “Appendix 1.” This will initiate the download of a compressed (pdf) archive that contains the file.

## Appendix 2. Significant genes of interest from the microarray analysis.

Selected genes met significance criteria (|fold change| > 2; FDR α<0.05) for CNTF treatment with SM as a control. These genes were then analyzed for their fold changes in the FBS group with SM as a control and again in CNTF with FBS as a control. The background color for each gene symbol cell corresponds to the location of the gene in the hierarchical cluster analysis presented in Figure 3. Asterisk (*) denotes significant fold change for that treatment comparison (|fold change| > 2; FDR α<0.05). To access the data, click or select the words “Appendix 2.” This will initiate the download of a compressed (pdf) archive that contains the file.
